# Effect of Supplemental Fixation on Fusion and Subsidence After Lateral Lumbar Interbody Fusion

**DOI:** 10.7759/cureus.82844

**Published:** 2025-04-23

**Authors:** Lauren C Levy, Jacob Greisman, Asham Khan, Mohamed A Soliman, Alexander O Aguirre, Jeffrey Mullin, John Pollina

**Affiliations:** 1 Neuosurgery, Massachusetts Institute of Technology, Cambridge, USA; 2 Neurosurgery, University at Buffalo Jacobs School of Medicine and Biomedical Sciences, Buffalo, USA; 3 Neurosurgery, Cairo University, Cairo, EGY

**Keywords:** fusion rates, lateral lumbar fusion, sagittal alignment, subsidence rates, supplemental fixation

## Abstract

Background

Lateral lumbar interbody fusion (LLIF) is currently popularized as an effective and safe approach to attain a lumbar fusion. The effect that supplemental fixation plays on fusion and subsidence rates after lateral lumbar surgery remains understudied. This study aims to directly compare fusion rates and subsidence rates in patients undergoing single-level lateral lumbar surgery with and without supplemental fixation for the treatment of degenerative disc disease with or without spondylolisthesis.

Methods

We included all single-level lateral interbody procedures for degenerative disc disease with or without spondylolisthesis. We excluded adult spinal deformity, infections, trauma, neoplastic pathology, and patients undergoing lateral interbody fusion of more than one level. Basic demographic data, lumbar lordosis, subsidence rates, and fusion rates were measured across the two groups.

Results

Forty-five (54.2%) patients underwent interbody placement alone without posterior supplemental fixation, while thirty-eight (45.8%) patients underwent percutaneous pedicle screw placement in addition to interbody placement. Only two patients underwent interbody placement at L1-2, none of whom received posterior fixation. Also, there was no significant difference in the mean change in lumbar lordosis (LL) angle between both groups (p=0.7). Furthermore, there was no significant difference in terms of the fusion (p=0.5) and subsidence (p=0.4) grades between both groups. The operative time was significantly longer in the supplemented fixation group (P<0.001). However, there was no significant difference in the hospital stay between both groups (p=0.7).

Conclusion

Supplemental fixation did not significantly affect fusion or subsidence rates in this study. Further studies are warranted to verify the results of this study.

## Introduction

Fusion procedures for degenerative spine disease are common and expensive, incurring approximately $14.1 billion in aggregate hospital costs in 2021 [1]. Minimally invasive surgery (MIS) is economically favorable relative to open surgery, with an average cost per quality-adjusted life year of $42,635 vs. $226,304 after one year [2]. Lateral lumbar interbody fusion (LLIF) is an increasingly popular MIS approach.

The LLIF is effective and safe. Fusion rates can approach 92% at one year postoperatively [3]. Unlike posterior approaches, the LLIF avoids manipulation of critical structures with known morbidity; it preserves posterior bony structures and posterior and anterior longitudinal ligaments and permits graft insertion without retraction of thecal sac and nerve roots [4]. Relative to other anterior approaches, the LLIF permits visualization through psoas manipulation, minimizing risks associated with great vessel mobilization [5]. Despite the strong association with positive radiographic and clinical outcomes, the risk of complications exists.

Cage subsidence refers to pathologic interbody cage depression into the adjacent vertebral endplate beyond the physiologic endplate remodeling, or “cage settling,” in response to biomechanical stress [6]. The degree of depression determines the grade [7]. Subsidence after LLIF is a known complication associated with significant morbidity that can warrant revision surgery [8]. Incidence can range from 10-20% [9]. Supplemental MIS posterior fixation with percutaneous pedicle screws can augment construct stability [10]. However, the impact on outcomes remains debated [11].

We aimed to retrospectively review patients with lumbar degenerative disease who underwent LLIF with and without posterior supplemental fixation.

## Materials and methods

The study was conducted at the Buffalo General Hospital, Buffalo, New York, United States. After obtaining Institutional Review Board approval, we conducted a retrospective chart review of patients who underwent LLIF for degenerative disease with or without spondylolisthesis between January 2014 and October 2016. A single surgeon performed all procedures at one academic institution and was assisted by an access surgeon. Patients undergoing multilevel fusions or with deformity, infectious, traumatic, or neoplastic pathology were excluded.

All patients were admitted postoperatively. Postoperative outpatient follow-ups with anteroposterior (AP) and lateral radiographs were scheduled at one week, four weeks, three months, six months, and one year postoperatively. CT indications included clinical concern for non-union and residual or recurrent symptoms. 

We used pedicle screws in patients that have high sacral slopes, are obese, are female, and have a smoking history. In the absence of high sacral slopes, pars defects, and multi-level interbodies, we typically perform unilateral fixation.

Lateral lumbar interbody fusion procedure

All patients underwent a lateral retroperitoneal transpsoas approach in the lateral decubitus position from the left side with a small break in the table. Localization with fluoroscopy was performed before a small linear incision across the flank. After opening the abdominal fascia, retroperitoneal finger dissection proceeds to the lateral aspect of the spine. We routinely stimulated our docking surface to monitor proximity to the lumbar plexus. The psoas is sequentially dilated and retracted, with or without shims, to maintain a clear field of view. A discectomy is carried out on the contralateral annulus, which is punctured with a Cobb elevator to further mobilize the disc space. The appropriately sized interbody cage with bone morphogenic protein allograft is selected to span the apophyseal ring, confirmed with AP fluoroscopy. 

If applicable, concomitant posterior fixation was performed the same day or as a staged procedure two days later with percutaneous pedicle screw placement with stereotactic navigation or robotic guidance.

Outcomes

Demographic data included age (years), sex, and body mass index (BMI; kg/m²). Operative duration (minutes), perioperative hospital length of stay (days; LOS), and interval duration to follow-up (months) were recorded. 

Primary outcomes included fusion and cage subsidence. Subsidence was measured according to the percentage loss of disc height devised by Marchi and colleagues [7] using lateral radiographs. Grade I is 0-24%; Grade II: 25-49%; Grade III: 50-74%; Grade IV: 75-100%. Fusion status was assessed with computer tomography (CT) scans, if available, and plain radiographs using the graded anterior scale published by Molinari et al. [12] Grade I: fused with remodeling and trabeculae present; Grade 2: graft intact, not fully remodeled, no lucency; Grade 3: graft intact, potential lucency at the top or bottom of graft; Grade 4: fusion absent with graft collapse or resorption.

Secondary outcome measurements included immediate postoperative change in coronal Cobb (CC) angle and lumbar lordosis (LL). LL was assessed on lateral radiographs and measured from the superior endplate of L1 to the endplate of S1. The CC angle was measured on AP radiographs by the angle formed by a line across the L1 superior endplate and the endplate of S1. Alignment parameters were measured on plain radiographs using the Surgimap software package (Surgimap, New York, NY, USA).

Statistical analysis

All statistical analysis was performed using the Statistical Analysis Software (SAS) package (SAS Institute Inc., Cary, NC, USA). Continuous variables were reported descriptively (mean ± standard deviation (SD), range) and compared with independent samples t-tests. Categorical variables were compared with chi-square tests. Changes in lumbar lordosis and CC angles were analyzed with logistic regression.

## Results

Eighty-three patients (mean age 57.6±10.5 years, mean BMI 30.6±5.2 kg/m², 50.6% female) were included, of whom 42 (50.6%) were female (Table 1). Three patients (3.6%) developed perioperative complications, including postoperative hematoma, seizure, and palpitations; all patients were discharged in stable condition (Table 2). Thirteen (15.7%) patients were lost to follow-up, 10 (76.9%) of whom received supplemental fixation, and three did not.

**Table 1:** Patient demographics and surgical characteristics. Independent samples t-tests were used to compare the means of continuous variables. Chi-square tests were used for categorical variables. BMI: body mass index; N/A: not applicable; LL: LL: lumbar lordosis; CC: coronal Cobb

Characteristics	Variable	All Patients (N=83)	Supplemental Fixation Group (N=38)	Non-supplemental Fixation Group (N=45)	Statistic	p-value
Demographics	Mean Age (years) ± SD	57.6 ± 10.5	56.3 ± 10.1	58.7 ± 10.7	T=-1.05	0.30
	Female (N, %)	42 (50.6%)	22 (57.9%)	20 (44.4%)	χ²(1)=1.464	0.23
	Mean BMI (kg/m²) ± SD	30.6 ± 5.2	30.2 ± 5.1	31.0 ± 5.3	T=-0.70	0.49
Pedicle screws	Unilateral (N, %)	N/A	24 (63.2%)	N/A		
	Bilateral (N, %)	N/A	14 (36.8%)	N/A		
Technique	Lateral Plating (N, %)	N/A	N/A	41 (91.1%)		
	Stand-Alone Cages (N, %)	N/A	N/A	4 (8.9%)		
Spine levels	L1-2 (N, %)	2 (2.4%)	0 (0%)	2 (4.4%)		
	L2-3 (N, %)	8 (9.6%)	3 (7.9%)	5 (11.1%)		
	L3-4 (N, %)	25 (30.1%)	9 (23.7%)	16 (35.6%)		
	L4-5 (N, %)	48 (57.8%)	26 (68.4%)	23 (51.1%)		
Perioperative factors	Mean Operative Time (min) ± SD	109.2 ± 57.9	156.2 ± 55.6	69.5 ± 11.7	T=9.44	<0.001
	Mean Length of Stay (days) ± SD	1.9 ± 1.0	2.0 ± 1.0	1.9 ± 1.1	T=-0.43	0.67
Alignment	Mean Preoperative LL Angle (degrees) ± SD	54.7 ± 11.9	55.6 ± 13.2	54.0 ± 10.8	T=0.60	0.55
	Mean Postoperative LL Angle (degrees) ± SD	52.5 ± 12.9	52.7 ± 12.4	52.3 ± 13.3	T=0.14	0.89
	Mean Change in LL Angle (degrees) ± SD	-2.2 ± 11.5	-2.9 ± 11.6	-1.6 ± 11.4	T=-0.51	0.61
	Mean Change in CC Angle (degrees) ± SD	-0.2 ± 3.7	-1.1 ± 3.4	0.4 ± 3.9	T=-1.87	0.07

**Table 2:** Surgical and radiographic outcomes. Chi-square tests were used to compare postoperative fusion grade and cage subsidence, and Fisher’s exact test for complications.

Characteristics	Variables	Supplemental Fixation Group (N=28)	Non-supplemental Fixation Group (N=42)	p-value
Postoperative fusion grade	Grade I (N, %)	20 (71.4%)	30 (71.4%)	0.92
	Grade II (N, %)	7 (25.0%)	8 (19.0%)	
	Grade III (N, %)	1 (3.6%)	2 (4.8%)	
	Grade IV (N, %)	0 (0%)	2 (4.8%)	
Cage subsidence	Any Subsidence (N, %)	17 (60.7%)	16 (38.1%)	0.05
	Grade I (N, %)	6 (21.4%)	11 (26.2%)	
	Grade II (N, %)	1 (3.6%)	3 (7.1%)	
	Grade III (N, %)	0 (0%)	2 (4.8%)	
Complications	Pelvic Hematoma (N, %)	1 (3.6%)	0 (0%)	0.41
	Seizures (N, %)	0 (0%)	1 (2.4%)	0.49
	Palpitations (N, %)	0 (0%)	1 (2.4%)	0.49

Supplemental fixation group 

**Figure 1:**
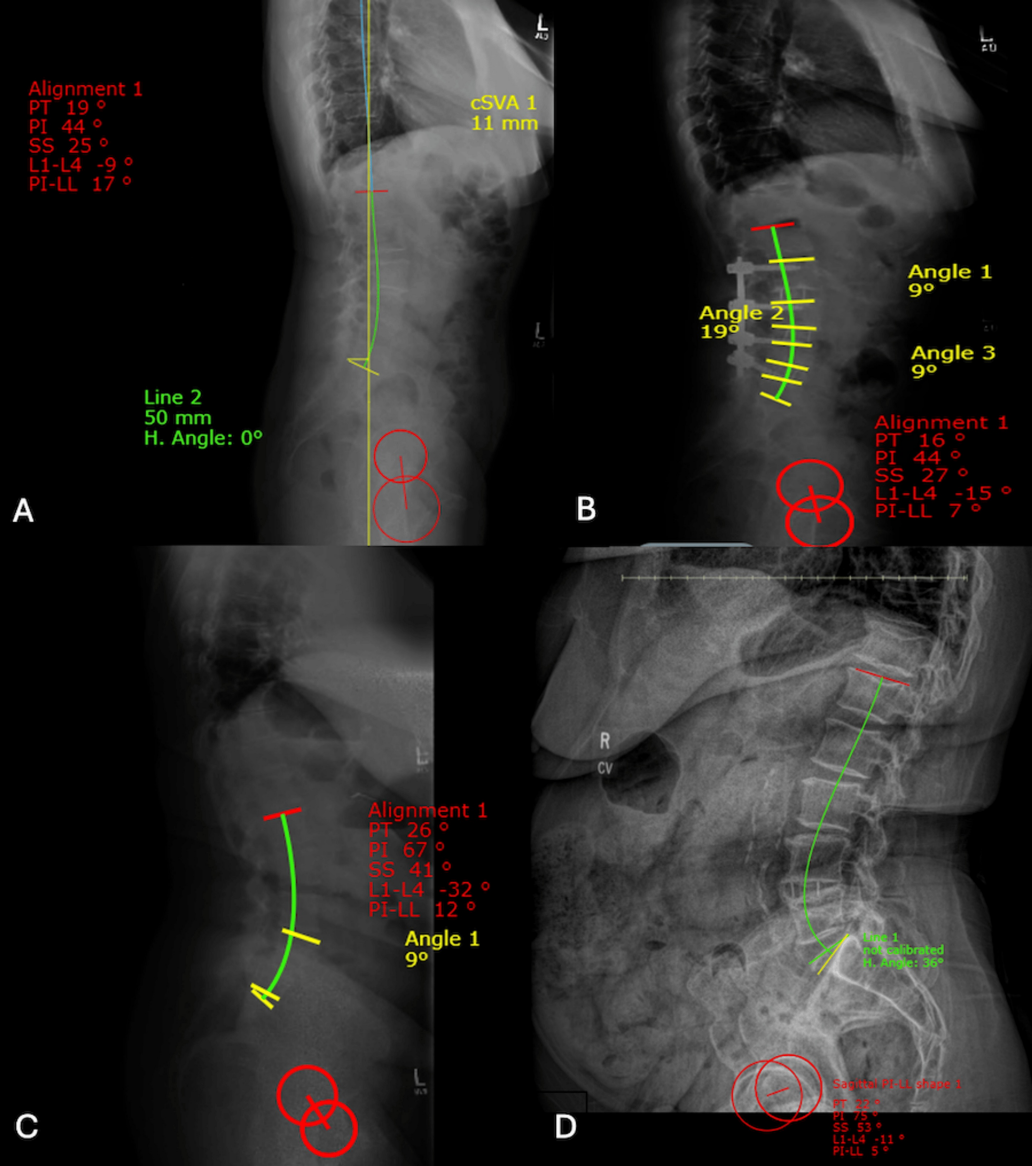
Illustrative lateral X-ray. A: preoperative of a patient in the supplemental fixation group; B: postoperative of the same patient in the supplemental fixation group; C: preoperative of a patient in the non-supplemental fixation group; D: postoperative of the same patient in the non-supplemental fixation group L: lumbar; LL: lumbar lordosis; PI: pelvic incidence; PT: pelvic tilt; SS: sacral slope

Thirty-eight (45.8%) patients received supplemental fixation. Twenty-four (63.2%) patients underwent unilateral pedicle screw placement, and 14 (36.8%) underwent bilateral pedicle screw placement. The mean operative time was 156.2±55.6 minutes (range, 58-386 minutes), and the mean LOS was 2±1 days (range, one to four days). The mean preoperative, postoperative, and change in LL angle was 55.6°±13.2°, 52.7°±12.4°, and -2.9°±11.6°, respectively, as shown in Figures 1A, 1B. The mean CC change was -1.1±3.4º. One patient developed a pelvic hematoma postoperatively; no other complications were reported.

Twenty-eight (73.7%) of 38 patients presented to the follow-up clinic. Interval duration from surgery ranged from two to 14 months (mean 5.8±3.4 months). The degree of postoperative fusion spanned grades I (N=20, 71.4%), II (N=7, 25.0%), and III (N=1, 3.6%). Cage subsidence occurred in 17 (60.7%) patients, including grade I (N=6, 21.4%) and grade II (N=1, 3.6%). 

Non-supplemental fixation group

Forty-five patients underwent fusion without posterior supplemental fixation. Lateral plating was used in 41 (91.1%) patients; stand-alone cages were used in four. The mean operative time was 69.5±11.7 minutes (range, 51-113 minutes), and the mean LOS was 1.9±1.1 days (range, one to six days). The mean preoperative, postoperative, and change in LL angle was 54°±10.8°,52.3°±13.3°, and -1.6°±11.4°, respectively, as shown in Figures 1C, 1D. The mean CC change was 0.4±3.9º. One (2.2%) patient developed postoperative seizures, and one (2.2%) developed palpitations; no other complications were reported.

Forty-two (93.3%) of 45 patients presented to the follow-up clinic. Interval duration from surgery ranged from two to 39 months (mean 9.5±8.7 months). Of the 42 patients with follow-up data, the degree of postoperative fusion spanned grades I (N=30, 71.4%), II (N=8, 19%), III (N=2, 4.8%), and IV (N=2, 4.8%). Cage subsidence occurred in 16 (38.1%) patients of varying severity, including grade I (N=11, 26.2%), grade II (N=3, 7.1%), and grade III (N=2, 4.8%).

Supplemental fixation versus non-supplemental fixation

Age, sex, BMI, LOS, and change in LL and CC angles do not significantly differ between groups (Table 1). The operative time was significantly longer in the supplemented fixation group (T score 9.44, p<0.001). Only two patients underwent interbody placement at L1-2, neither of whom received posterior fixation. Rates and degrees of subsidence χ²(1)=3.84, p=0.05) and fusion (χ²(3)=0.02, p=0.92) and subsidence did not significantly differ.

## Discussion

LLIF is an increasingly popular approach to treating degenerative lumbar disease due to favorable radiographic and clinical outcomes [11,13-15]. While supplemental posterior fixation adds construct stability, the clinical impact on fusion and subsidence rates after LLIF is less clear [16,17]. Our results do not demonstrate a statistically significant reduction in subsidence or improvement in fusion associated with supplemental fixation. 

Although the supplemental fixation group had greater overall subsidence, the correlation was not significant and likely subject to significant confounds, warranting consideration in future studies. Patient and operative factors that may influence subsidence rates warrant consideration in future studies, such as concomitant osteoporosis and cage size [7,18]. Reduced bone mineral density reduces the bone strength required to counter the mechanical, compressive forces driving subsidence [18]. Furthermore, wider cages span a greater surface area of the apophyseal ring, which contributes significant structural integrity [18].

Fusion rates after LLIF range from 85-100% [8,11,19]. Our 95.2% fusion rate at 9.5 months follow-up aligns with previous studies. While literature exists reporting enhanced fusion after posterior fixation, patient selection is critical [20].

LLIF procedures can improve and maintain both global and segmental LL [11,21,22]. However, the effect of supplemental fixation remains debated [11,21,22,23]. Our results failed to demonstrate a significant difference in alignment parameters between the two groups. Larger, prospective studies are warranted. 

The posterior supplemental fixation group demonstrated significantly increased operative time. This is expected, as additional instrumentation increases the steps in the procedure and is consistent with previous studies [23]. Increased procedural duration carries its own set of risks related to increased time under anesthesia and potentially increased blood loss. However, increased operative time did not significantly correlate with LOS. Performing staged surgeries likely contributed to these findings [24].

The use of supplemental fixation was up to the discretion of the senior author of this study; however, the factors that affect the decision-making are usually advanced spondylolisthesis, poor bone quality, and severe canal stenosis that requires posterior decompression. 

Limitations

Our study was limited by its retrospective nature and small sample size. Further, within-group variation, such as stand-alone cages versus lateral plating in the group without supplemental fixation and unilateral versus bilateral pedicle screw placement in the group with supplemental fixation, may have impacted our results and warrants further investigation.

## Conclusions

LLIF is a minimally invasive technique associated with positive radiographic and clinical outcomes. Although our results do not demonstrate significant radiographic or clinical differences between groups that did or did not undergo supplemental posterior fixation. However, LLIF without supplemental posterior fixation demonstrated a significantly shorter operative time compared to the supplemented fixation group. However, supplemental fixation did not result in a statistically significant difference in subsidence rates, fusion rates, or sagittal alignment changes. These findings suggest that whether routine posterior fixation may not be necessary for all LLIF procedures remains inconclusive, though patient-specific factors must be considered. Larger, prospective studies are needed to further elucidate the impact of supplemental fixation on long-term outcomes and to refine patient selection criteria for its use.
